# Enhancing Permanent Magnet Sliding Bearings Through Multi-Layer Yoke for Minimized Magnetic Leakage

**DOI:** 10.3390/ma19030642

**Published:** 2026-02-06

**Authors:** Yong Liu, Haitao Zhao, Jixing Li, Lei Wu, Yang Xia

**Affiliations:** 1 College of Nuclear Science and Technology, Naval University of Engineering, Wuhan 430033, China; 2 School of Mechanics and Aerospace Engineering, Dalian University of Technology, Dalian 116024, China; 3 State Key Laboratory of Marine Thermal Power Technology, Wuhan Second Ship Design and Research Institute, Wuhan 430205, China

**Keywords:** permanent magnet bearings, multi-layer yoke structure, magnetic leakage, magnetic field analysis

## Abstract

To mitigate the potential adverse effects of magnetic flux leakage from permanent-magnet sliding bearings on human health and the environment, this study proposes a leakage-suppressed design based on a multi-layer yoke configuration. The magnetic performance of the bearing was systematically investigated using finite element method (FEM) simulations. The results demonstrate a pronounced reduction in magnetic leakage when replacing a conventional single-layer yoke with an optimized multi-layer yoke structure. Targeted design refinements, including optimization of both the number and angular span of magnetic rings, as well as tuning of the yoke thickness, further enhance the effectiveness of the leakage-suppression strategy. The proposed multi-layer yoke configuration preserves both the magnetic force and the load-carrying capacity of the magnetic bearing, while concurrently providing a viable theoretical and engineering basis for the design and structural optimization of leakage-controlled permanent-magnet bearings.

## 1. Introduction

Magnetic levitation bearings, commonly referred to as magnetic bearings, support rotors by means of magnetic forces acting between the stator and the rotor, thereby partially or completely eliminating mechanical contact. This feature renders them particularly suitable for high-speed, vacuum, and ultra-clean operating conditions, as they do not require lubrication and are not subject to frictional losses or mechanical wear [1]. Magnetic bearings are generally categorized into active, passive, and hybrid types according to the source and regulation of the magnetic forces [2]. Passive magnetic bearings utilize rare-earth permanent magnetic materials, thereby enabling permanent magnet bearings to provide support for the rotating shaft. By appropriately configuring the arrangement of permanent magnet rings, they generate stabilizing forces that can substantially reduce friction. Passive magnetic bearings offer several notable advantages, including low energy consumption, compact structural dimensions, relatively low cost, ease of maintenance, high operational reliability, and the absence of a requirement for an active control system.

The application scope of permanent magnet bearings is extensive, encompassing a wide range of industrial sectors, including eddy current couplers [3], high-speed compressors [4], wind turbines [5,6], miniature axial-flow pumps [7], flywheel energy storage systems (FESS) [8,9], ventricular assist devices and other heart pumps [10], as well as long-distance power transmission systems [11]. Lemarquand et al. [12,13] demonstrated that the load-carrying capacity and stiffness of such bearings are essentially independent of the specific magnetization pattern. On this basis, advanced configurations, such as stacked magnet structures, have been proposed to further enhance bearing stiffness. For example, Samanta [14] developed a configuration in which aluminum bearing rings are embedded into permanent magnet blocks, and experimental investigations have confirmed that this approach increases both load capacity and dynamic stability.

Subsequent research by Bekinal et al. [15] has concentrated on characterizing the nominally frictionless behavior and high-precision design requirements of permanent magnet bearings. Their work highlights the substantial design flexibility of these systems with respect to geometric configuration, analytical and numerical modeling methodologies, and recent technological developments that address intrinsic instability issues and facilitate hybridization with other bearing types over a broad range of operating speeds. Furthermore, Haidl and Buchroithner [16] describe the development of a cost-effective, low-loss bearing architecture, which is closely aligned with the design principles and performance objectives of passive magnetic bearing systems. In the specific context of flywheel energy storage, new design strategies—such as the analysis and optimization of high-efficiency axial-suspension permanent magnet sliding bearings—have been proposed [17], demonstrating the practical viability and performance advantages of permanent magnet bearings in real-world applications.

Within the field of marine bearing design, multiple innovative methodologies employing magnetic bearings have been proposed to improve operational performance and efficiency. Li [18] introduced a magnetic composite bearing incorporating a Halbach permanent magnet array. This configuration substantially decreases contact stress and structural deformation relative to conventional bearing designs. Building on magnetic support technology, He [19] developed a novel sliding bearing that integrates a permanent magnet-generated support force. This design effectively improves lubrication conditions, increases bearing load-carrying capacity, and enhances the overall dynamic stability of the system.

Employing a fully coupled fluid–solid–magnetic multiphysics model, Li [20] proposed a magnetic–fluid dual-floating bearing configuration. The study demonstrated that a Halbach non-integral magnetic circuit arrangement can significantly enhance the load-carrying capacity of the bearing. Wang [21] developed a magnetic–fluid dual-suspension bearing with high load capacity, in which the combined effects of hydrodynamic pressure and permanent-magnet repulsion yield superior performance. B. Wang [22] further introduced a magnetic–fluid dual-suspension elastic-support sliding bearing. Relative to conventional water-lubricated bearings, this design improves load capacity under heavy and fluctuating operating conditions by incorporating magnetic forces.

Zhou et al. [23] formulated a theoretical model for Halbach array-based permanent magnetic bearings (PMB) and established a dynamic model of a PMB–rotor system subjected to base excitation. Using the root-locus method, they investigated the influence of key parameters on system stability and thereby provided a theoretical foundation for the engineering application of PMBs. Li [24] developed PMB prototypes for flywheel batteries in new-energy vehicles, constructed analytical and dynamic models, performed experimental validation, and carried out optimization via the NSGA-II algorithm (Pareto frontier), resulting in a low-cost, high-load optimized PMB design. Cheng et al. [25] proposed a permanent-magnet/foil hybrid bearing to address the low load-carrying capacity and start–stop friction issues inherent to foil bearings. They formulated a corresponding dynamic model, systematically investigated the influence of key design parameters through trajectory-based analyses, and demonstrated that the hybrid bearing attenuates rotor eccentricity while promoting a more favorable load distribution.

Ge et al. [26] developed a novel modular permanent-magnet-biased magnetic bearing aimed at overcoming limitations associated with conventional design and manufacturing practices. The proposed configuration was investigated and validated through an equivalent magnetic circuit model in combination with finite element analyses conducted in ANSYS Maxwell 2020R2. The results demonstrate that the modular architecture can be flexibly assembled to meet diverse operating requirements, and that a single module is capable of generating a supporting force of approximately 500 N at a current of 3 A. Cheng et al. [27] resolved the issues of axial instability and stiffness deviation in radial permanent magnetic bearings by employing equivalent magnetic charge theory, a spherical hinge structure, and a dedicated experimental test rig. They quantified the magnetic forces involved, successfully achieved magnetic suspension of an 18 kg rotor, and experimentally determined the stiffness characteristics. Supreeth et al. [28] employed finite element analysis to determine the working reluctance length, identified the 10-HN B–R configuration as providing the lowest prediction error, and subsequently optimized the system using a MATLAB R2021-implemented Bonobo optimization algorithm. The optimized design was further validated through COMSOL Multiphysics simulations, thereby substantiating the efficacy of artificial neural network (ANN) models for the prediction and performance enhancement of eddy current dampers (EDB). Margarit et al. [29] reviewed recent advances in the modification of SmCo_5_-type permanent magnets, proposing targeted chemical substitutions—Fe/Ni for Co and Ce/La for Sm—to mitigate dependence on critical and costly raw materials. They further demonstrated that ab initio and density functional theory (DFT) calculations provide a robust framework for accurately predicting both the thermodynamic stability of rare-earth–transition-metal (RE–TM) intermetallic compounds and their intrinsic magnetic properties. Amit et al. [30] developed a process for the recovery of Nd and Pr from end-of-life Nd–Fe–B permanent magnets, thereby demonstrating the critical role of rare-earth magnet recycling in supporting the green energy transition, enhancing the security and resilience of rare-earth supply chains, and evidencing the economic competitiveness of short-loop recycling pathways relative to conventional metallurgical processes. Tomaž et al. [31] investigated the effects of thermal demagnetization in air and vacuum on the coating stability and remagnetization performance of Nd–Fe–B magnets, and identified suitable protective coatings through systematic corrosion testing.

To mitigate the potential effects of electromagnetic field exposure on the human nervous system, the establishment of well-defined exposure limits in relevant environments is essential. Widely adopted reference standards for electromagnetic protection include the guidelines issued by the International Commission on Non-Ionizing Radiation Protection (ICNIRP) [32,33], which recommend a minimum threshold of 0.5 mT for individuals with ferromagnetic bodily implants or those using medical devices. In addition, in 2002, the Institute of Electrical and Electronics Engineers (IEEEs) issued standards regulating human exposure to radiofrequency electromagnetic fields in the 0–3 kHz frequency range [34], in which short-term contact limits for controlled environments are specified. In a related context, Contessa et al. [35] analyzed the challenges and operational issues associated with the implementation of Directive 2013/35/EU on the protection of workers from EMF exposure, with particular emphasis on interpretative and procedural difficulties in industrial settings. Their work outlines methodologies for addressing overexposure scenarios and proposes practical strategies to achieve and demonstrate regulatory compliance.

As research advances and the understanding of electromagnetic field (EMF) exposure deepens, the implementation of robust electromagnetic protection strategies is becoming increasingly critical for the protection of human health and safety. International standards and guidelines, such as those issued by the ICNIRP and the IEEE provide explicit criteria for the design and operation of electromagnetic devices. These documents highlight the necessity of rigorously assessing and controlling EMF emissions, particularly in high-performance applications such as magnetic levitation systems. They further emphasize the importance of embedding electromagnetic characteristics and exposure considerations into the design and development of novel bearing configurations and other magnetically actuated devices.

Within this framework, the present study proposes an innovative multi-layer yoke configuration for permanent magnet sliding bearings. The design objective extends beyond conventional goals of magnetic circuit optimization and reduction in magnetic flux leakage; it also explicitly incorporates EMF protection requirements into the engineering process. By integrating these protective considerations at the design stage, the proposed approach seeks to simultaneously improve device performance and operational safety, thereby facilitating compliance with increasingly stringent EMF exposure standards.

This study proposes a multi-layer architecture for permanent magnet sliding bearings. Specifically, a multi-layer yoke configuration is developed that significantly suppresses magnetic flux leakage while maintaining the bearing’s load-carrying capacity. The proposed multi-layer yoke configuration preserves both the magnetic force and the load-carrying capacity of the magnetic bearing, while concurrently providing a viable theoretical and engineering basis for the design and structural optimization of leakage-controlled permanent-magnet bearings. Through comprehensive magnetic circuit analysis combined with finite element simulations, the work delivers optimized magnetic circuit design schemes and concomitant refinements of the overall bearing architecture. The results highlight the suitability of the proposed bearing structure for high-performance magnetic levitation systems, support the systematic optimization of existing bearing technologies, and open new avenues for research and innovation in magnetic bearing design.

## 2. The Structure of Permanent Magnet Sliding Bearings

The structural configuration and operational principles of permanent magnet sliding bearings are described as follows. The bearing assembly is principally composed of two subsystems: a conductive rotor and a stator. The conductive rotor consists of a magnetic sleeve and a drive shaft, whereas the stator is formed by an array of permanent magnets, magnetic yokes, and spacers [18,19,20]. The magnetic sleeve is rigidly mounted on the drive shaft, and when the motor is energized, the conductive rotor rotates within the magnetic field established by the stator.

**Figure 1:**
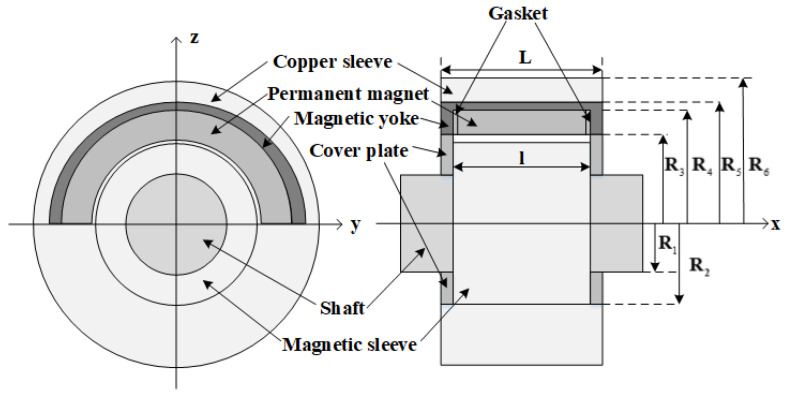
Geometrical configuration of permanent magnet-based sliding bearings.

Figure 1 illustrates the fundamental configuration of a permanent magnet sliding bearing, emphasizing the presence of an air gap between the permanent magnet stator and the electrically conductive rotor. The resulting magnetic force can be adjusted in real time by varying the thickness of this air gap. A magnetic yoke, fabricated from a material with high magnetic permeability, is positioned along the upper, frontal, and rear boundaries of the permanent magnet array. Its primary function is to guide and confine the magnetic flux generated by the permanent magnet ring, thereby significantly reducing magnetic flux leakage and enhancing the localization and intensity of the magnetic field. Furthermore, a cover plate composed of a soft magnetic material is incorporated to additionally suppress the leakage of magnetic flux propagating along the air gap toward the axial ends of the bearing, which is highly beneficial for minimizing the overall magnetic flux loss.

The characteristic geometrical parameters of the conductive rotor segment are summarized in Table 1, whereas the geometrical parameters of the stator segment are compiled in Table 2.

The axial width of the permanent magnet ring is specified as 30 mm, with Neodymium– Iron–Boron (NdFeB) selected as the magnet material. The principal magnetic properties of the chosen grade N52 include a remanent flux density of 1.44 T, an intrinsic coercivity of $H_{c}=954.93$ kA/m, a maximum magnetic energy product of $BH_{\max}=395$ kJ/$m^{3}$, and a maximum recommended operating temperature of 60 ℃. He et al. [20,21] experimentally and numerically demonstrated that Halbach magnetization configurations substantially enhance load-carrying capacity, thereby providing the rationale for their adoption in the present study.

Silicon steel is employed as the magnetic yoke material. This selection is based on its classification as a soft magnetic alloy, characterized by high magnetic permeability and low hysteresis loss, which enables efficient magnetic flux conduction and facilitates rapid demagnetization upon removal of the external field. Polytetrafluoroethylene (PTFE) is chosen as the bearing pad material. This polymer exhibits an exceptionally low coefficient of friction, pronounced non-adhesive behavior, and high corrosion resistance attributable to its excellent chemical inertness, rendering it suitable for long-term operation in aggressive environmental conditions. Copper is selected for the bearing casing due to its favorable tribological properties, good corrosion resistance, and its ability to sustain elevated loads and rotational speeds, thereby improving the overall performance and service life of the bearing assembly.

In the design of the permanent-magnet sliding bearing, positioning the magnetic rings above the non-load-bearing region enables the formation of a controlled air gap between the rotor and the permanent-magnet array. This arrangement establishes a closed magnetic circuit consisting of the permanent magnets, the rotor, and the magnetic yoke. Within this system, the interaction between the permanent-magnet array on the stator and the rotor generates an upward magnetic force that partially compensates for the gravitational load of the shaft system. Simultaneously, the lower bearing pad provides mechanical support to the bottom of the shaft system, operating in conjunction with the magnetic ring to achieve accurate axial and radial positioning as well as enhanced dynamic stability. This integrated magneto-mechanical design improves the overall operational performance of the bearing and contributes to a significant extension of its service life.

## 3. Investigation of the Magnetic Field Characteristics of Permanent-Magnet Linear Bearings

### 3.1. Magnetic Circuit

**Figure 2:**
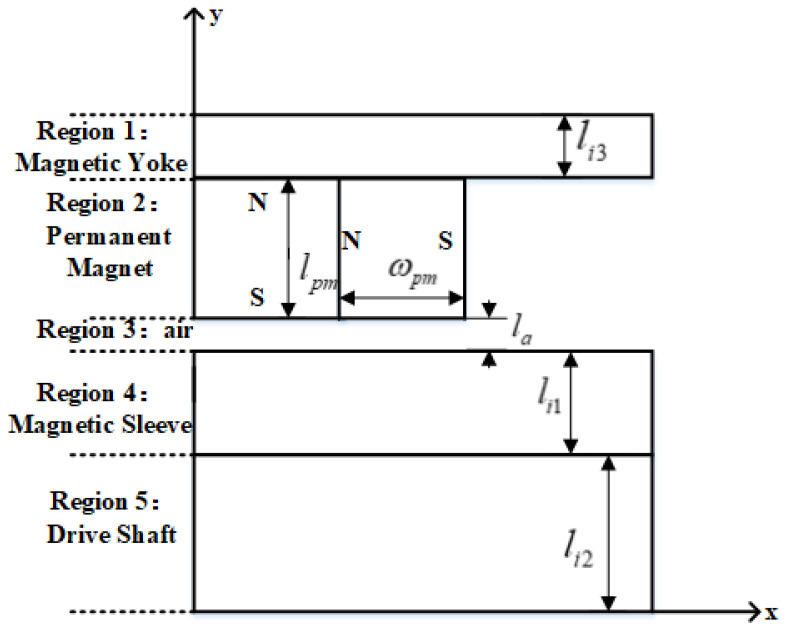
Two-dimensional theoretical model of a permanent magnet bearing.

**Figure 3:**
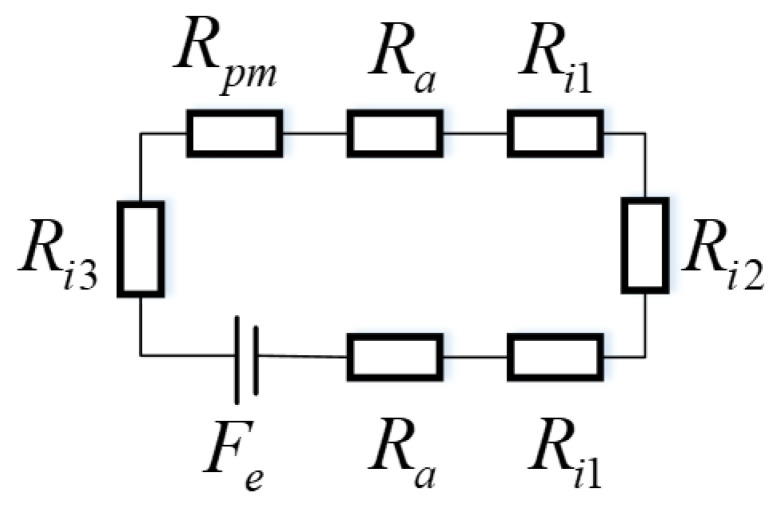
Simplified equivalent magnetic circuit model for the main flux path.

Based on the fundamental structural configuration shown in Figure 1, a reduced two-dimensional model of the permanent magnet bearing can be established, as presented in Figure 2. The actual magnetic field distribution in the bearing is complex, involving structural inhomogeneities, such as the gaskets and Halbach array segments, and significant flux branching. However, an equivalent magnetic circuit is intended as a simplified lumped-parameter model focusing exclusively on the main magnetic flux path. On this basis, by performing a magnetic circuit analysis, an equivalent magnetic circuit representation is formulated, as illustrated in Figure 3. This equivalent circuit accounts exclusively for the main magnetic flux and neglects the contribution of leakage flux. By applying the magnetic circuit formalism, the magnetic flux density can subsequently be evaluated, under the assumption that eddy current effects are negligible.

In Figure 2, $l_{pm}$ denotes the thickness of the permanent magnet, $\omega_{pm}$ denotes the width of the permanent magnet, $l_{a}$ denotes the air-gap thickness, $l_{i1}$ denotes the thickness of the magnetic sleeve, $l_{i2}$ denotes the thickness of the shaft, and $l_{i3}$ denotes the thickness of the magnetic yoke.

In Figure 3, $F_{e}$ denotes the internal magnetomotive force source associated with a pair of magnetic poles, $R_{a}$ represents the magnetic reluctance of the air gap, $R_{pm}$ corresponds to the magnetic reluctance of a magnetic pole, $R_{i1}$ indicates the magnetic reluctance of the magnetic sleeve, $R_{i2}$ denotes the magnetic reluctance of the shaft, and $R_{i3}$ represents the magnetic reluctance of the magnetic yoke. To facilitate the subsequent analytical derivation, the mathematical model employed in this study is established under the following assumptions:

(1) The analysis neglects the contribution of the induced magnetic field arising from eddy current phenomena.
(2) End effects and magnetic saturation effects on the magnetic field are omitted from the present formulation.
(3) The magnetic permeability of all materials is assumed to remain constant over the entire calculation and is considered invariant with respect to external influences.

### 3.2. Magnetic Flux Density and Magnetic Force

Within the structural configuration of a permanent magnet bearing, the permanent magnet serves as a stable and reproducible source of magnetic flux, thereby guaranteeing a consistent and sufficiently high magnetic force. By exploiting the intrinsic material properties and magnetization characteristics of the permanent magnet, the initial magnetomotive force can be determined with a high degree of accuracy.

(1) $$F=H_{c}l_{pm}$$

$H_{c}$ represents the coercivity of the permanent magnet.

According to the data presented in Figure 3, the magnetic reluctance of the permanent magnet can be expressed as follows:

(2) $$R_{pm}=\frac{l_{pm}}{\mu_{0}\mu_{r}\omega_{pm}L}$$

In this expression, $\mu_{0}$ denotes the magnetic permeability of free space (vacuum), and $\mu_{r}$ represents the relative magnetic permeability of the permanent magnet ring, defined as $\mu_{r}=-\frac{B_{r}}{\mu_{0}H_{c}}$, where $B_{r}$ is the remanent magnetic flux density and $H_{c}$ is the coercive field strength. The symbol *L* corresponds to the axial length of the permanent magnet. The effective magnetomotive force [36] is given by

(3) $$F_{e}=k_{0}F\frac{R_{pm}}{R_{pm}+R_{l}}$$

In this expression, $k_{0}$ denotes a correction factor that accounts for the actual operating conditions of the magnetic circuit, including variations in magnetic reluctance and the spatial distribution of magnetic flux. Its value may exhibit slight variations depending on the specific application scenario. The magnetic reluctance of the air gap is given by

(4) $$R_{a}=\frac{l_{a}}{\mu_{0}\mu_{a}\omega_{pm}L}$$

$\mu_{a}$ denotes the magnetic permeability of air. The magnetic reluctance of the magnetic sleeve is given by

(5) $$R_{i1}=\frac{l_{i1}}{\mu_{0}\mu_{i1}\omega_{pm}L}$$

The magnetic reluctance of the shaft is

(6) $$R_{i2}=\frac{l_{i2}}{\mu_{0}\mu_{i2}\omega_{pm}L}$$

The magnetic reluctance of the yoke is

(7) $$R_{i3}=\frac{l_{i3}}{\mu_{0}\mu_{i3}\omega_{pm}L}$$

On the basis of the equivalent magnetic circuit depicted in Figure 3 and the effective magneto-motive force defined in Equation (3), the corresponding effective magnetic flux is determined as follows:

(8) $$\phi =\frac{F_{e}}{2R_{a}+2R_{i1}+R_{i3}+R_{i2}+R_{pm}}$$

Consequently, the corresponding effective magnetic induction intensity can be expressed as

(9) $$B=\frac{\phi }{\omega_{pm}L}$$

In the computation of the magnetic force generated by the magnetic field of a permanent magnet, several methodologies may be adopted, including the equivalent magnetic charge method [37] and the virtual work principle method [38]. In the present study, the Maxwell stress tensor method [39] is employed to evaluate the attractive force exerted by the permanent magnet array on the rotor. For a singly connected region devoid of free currents, it is known that

(10) ∇×H=0

The magnetic scalar potential φ is defined as follows [39]:

(11) H=−∇φ

By appropriately substituting the relevant field quantities into Maxwell’s equations, the Maxwell stress tensor can be formulated in the following manner [40].

(12) $$T_{m}=-\mu \frac{H.H}{2}I+\mu H⊗H$$

Here, **I** denotes the identity tensor, and μ represents the magnetic permeability.

By applying Stokes’ theorem to a closed boundary Γ, which encloses the volume *V* and intersects the surface *S*, the resultant magnetic force $F_{m}$ acting on this volume can be determined. This total magnetic force is obtained by integrating the divergence of the Maxwell stress tensor $T_{m}$ over the volume *V*, as expressed below:

(13) $$F_{m}=\int_{V}\nabla \left(T_{m}\right)dV=\oint_{S}T_{m}.n_{1}ds=\oint_{\Gamma }\left(-\frac{\mu_{0}}{2}H^{2}n_{2}+\mu_{0}H_{n}H\right)d\Gamma$$

In this expression, $n_{1}$ denotes the unit normal vector to the surface *S*, $n_{2}$ represents the outward-pointing local normal vector along the boundary Γ, and $H_{n}$ designates the component of the magnetic field H in the direction normal to the surface.

### 3.3. Magnetic Medium Theory

#### 3.3.1. The Boundary Conditions for Magnetic Media

When a magnetic field transitions from one material medium to another, for instance, from air into a ferromagnetic medium, the components of the field are subject to well-defined electromagnetic boundary conditions that follow directly from Maxwell’s equations and the associated constitutive relations. To maintain physical and mathematical continuity at the interface, particular components of the magnetic field vectors must satisfy the following requirements:

(1) Continuity of the normal component of the magnetic flux density
  The normal component of the magnetic flux density remains continuous across a material interface. This implies that the net magnetic flux crossing the boundary is conserved. Mathematically, this condition is expressed as
  (14) $$B_{1n}=B_{2n}$$
  In this expression, $B_{1n}$ and $B_{2n}$ denote the components of the magnetic flux density vector that are normal to the interface, evaluated within the magnetic media on the respective sides of the boundary.
(2) Continuity of the tangential component of the magnetic field intensity
  The tangential component of the magnetic field intensity is likewise continuous across the interface between two media. This condition can be expressed mathematically as
  (15) $$H_{1t}=H_{2t}$$
  In this expression, $H_{1t}$ and $H_{2t}$ denote the tangential components of the magnetic field intensity within the magnetic media on the respective sides of the boundary.

#### 3.3.2. The Refraction of Magnetic Induction Lines

**Figure 4:**
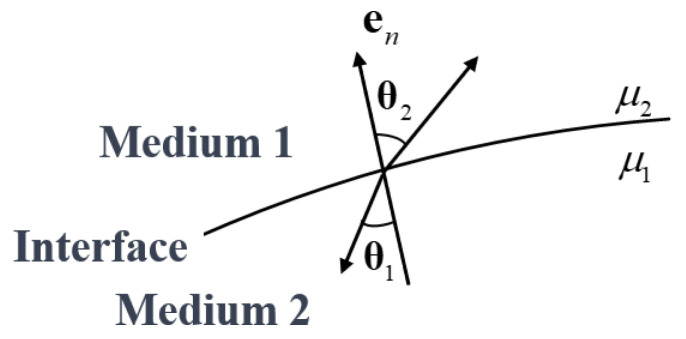
Refraction of magnetic field lines through the interface between two mediums.

When magnetic field lines propagate from one medium to another characterized by a different magnetic permeability, they undergo a refraction process analogous to the refraction of light rays at interfaces between optical media, as illustrated in Figure 4. This magnetic refraction is governed by, and can be quantitatively described through, the electromagnetic boundary conditions imposed on the magnetic field at the interface.

(16) $$\left\{\begin{array}{cc} B_{1n}=B_{1}\cos\theta_{1} & B_{2n}=B_{2}\cos\theta_{2}\\ H_{1t}=H_{1}\sin\theta_{1} & H_{2t}=H_{2}\sin\theta_{2} \end{array}\right.$$

Denoting the magnetic permeabilities of medium 1 and medium 2 by $\mu_{1}$ and $\mu_{2}$, respectively, we obtain

(17) $$\frac{\tan\theta_{1}}{\tan\theta_{2}}=\frac{\mu_{1}}{\mu_{2}}$$

**Figure 5:**
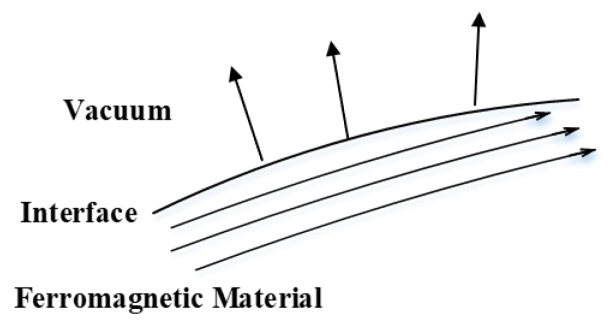
Refraction of magnetic field lines between ferromagnetic medium and vacuum region.

The aforementioned relation indicates that the ratio of the tangents of the angles formed by the magnetic induction lines with the normal to the interface is equal to the ratio of the magnetic permeabilities of the two media. When medium 2 is vacuum and medium 1 is a material with high magnetic permeability, as illustrated in Figure 5, the magnetic induction lines are predominantly confined within the ferromagnetic body. As the magnetic permeability of the ferromagnetic medium increases, the angle θ between the magnetic induction lines and the interface normal also increases, tending toward a configuration in which the induction lines are nearly parallel to the surface. As a consequence, the magnetic flux penetrating into the vacuum region is reduced, which enhances the suppression of magnetic field leakage and improves the effectiveness of magnetic shielding.

#### 3.3.3. Magnetic Shield

**Figure 6:**
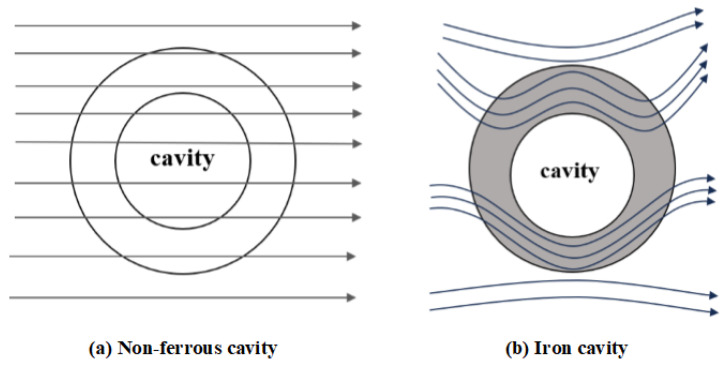
Magnetic shield phenomena.

The operational principle of magnetic shielding is fundamentally based on the concept of parallel magnetic circuits. In the configuration under consideration, the outer wall of the ferromagnetic shell and the vacuum inside the enclosed cavity together constitute a pair of parallel magnetic paths. Given that the magnetic permeability of a vacuum is approximately unity, whereas the relative magnetic permeability of the iron shell can attain values on the order of 10^3^ or higher, the magnetic reluctance of the cavity is substantially greater than that of the shell wall. As a result, the majority of magnetic flux preferentially propagates along the inner wall of the iron shell, as illustrated in Figure 6b. To further enhance the effectiveness of the magnetic shielding, a multi-layered ferromagnetic shell architecture may be employed, thereby progressively reducing the magnetic flux penetrating into the interior cavity.

By exploiting the principle of magnetic shielding and rigorously satisfying the associated boundary conditions, the introduction of multiple layers of magnetic yoke iron outside the permanent magnet can substantially reduce magnetic flux leakage from the exterior of the bearing. This strategy effectively enhances the overall efficiency of the magnetic shielding system.

In planar geometries or simple magnetic circuits, splitting a yoke into multiple layers of identical material without an air gap should theoretically yield a magnetic reluctance path identical to that of a single solid block. In such general cases, the flux propagation would indeed remain unchanged. In practical engineering applications, it is also important to note that while the multi-layer yoke has no macroscopic air gap, and it functions similarly to a laminated core typical of silicon steel applications. Such multi-layer structures are analogous to laminated cores, where microscopic inter-laminar insulation exists even in the absence of a macroscopic air gap. This structure introduces a high reluctance path in the radial direction perpendicular to the layers, thereby effectively forcing the magnetic flux to propagate tangentially along the yoke layers. The interface between layers serves to inhibit radial magnetic flux penetration, thereby guiding the magnetic field lines tangentially along the curvature of the yoke. This guiding effect reduces the leakage flux density in the external space.

While the single-layer and multi-layer structures behave similarly when the arc angle is small, the behavior diverges significantly as the angular span increases. The specific focus of the present work is on improving the magnetic shielding of a composite bearing using a curved, arc-shaped stator configuration, rather than a simple planar magnetic circuit. Simulation results in next section indicate that the geometric angular span of the yoke plays a decisive role in how the magnetic flux is distributed and confined. Specifically, for the bearing design where the magnetic yoke encompasses an angle of $180^{\circ }$ or greater, the multi-layer configuration demonstrates a superior capability in guiding magnetic flux lines tangentially along the curvature.

## 4. Influence of Structural Optimization and Multi-Layer Magnetic Yokes on Magnetic Leakage

### 4.1. Multi-Layer Magnetic Yoke

**Figure 7:**
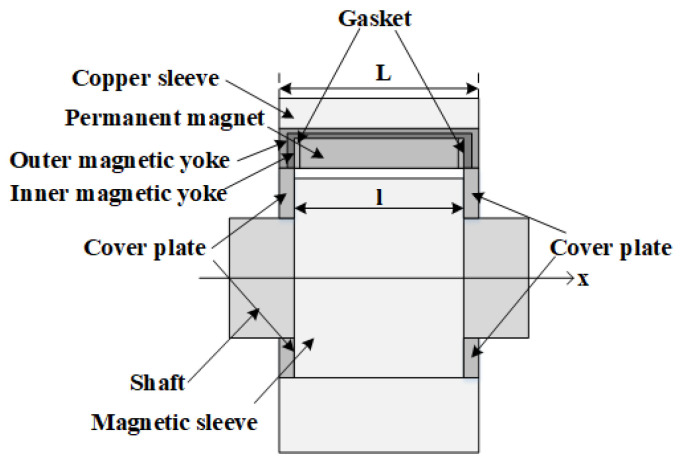
Multi-layer magnetic yoke diagram.

As discussed in the magnetic medium theory in Section 3.3, multi-layer magnetic yokes provide superior suppression of magnetic leakage relative to a single-layer configuration. In the present design, the original single-layer magnetic yoke with a thickness of 10 mm is reconfigured into a double-layer yoke, where each layer has a thickness of 5 mm and no gap is present between the layers. This modification yields the two-dimensional geometry illustrated in Figure 7. During the transition from a single-layer to a double-layer yoke, the magnetic force remains nearly unchanged, implying that the load-carrying capacity of the bearing is essentially unaffected.

#### 4.1.1. Comparison of Single-Layer and Multi-Layer Magnetic Yokes

**Figure 8:**
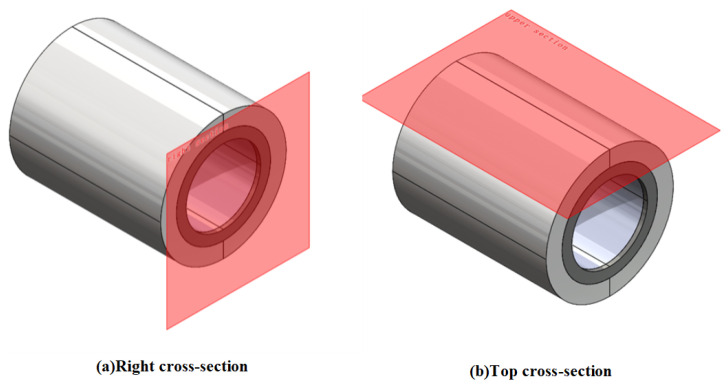
The view of cross-sections for the examination of magnetic flux density.

Li et al. [18] quantifies the degree of magnetic leakage by evaluating the maximum magnetic flux density on the upper surface of the magnetic yoke. In the present study, the magnetic leakage of the permanent magnet array is quantified by the maximum magnetic flux density measured at a position 10 mm from the right lateral surface and 10 mm from the upper surface of the magnetic yoke, as illustrated in Figure 8.

The magnetic field analysis is performed using the multiphysics simulation software COMSOL 6.0. The simulation model comprises a magnetic sleeve, rotating shaft, permanent magnet rings, yoke, spacers, and other components. The specific structural dimensions are presented in Table 3.

The axial width of a single permanent magnet ring is 30 mm. The selected material is Neodymium–Iron–Boron (NdFeB, Grade N52), with the following key parameters: Remnant Flux Density of 1.44 T, Coercivity of 954.93 kA/m, Maximum Energy Product of 395 ${\mathrm{kJ}/m}^{3}$, and a maximum operating temperature not exceeding 60 ℃. The materials and their corresponding relative permeability ($\mu_{r}$) for each component are listed in Table 4.

The mesh size is strictly configured according to the pre-set “Extra Fine” level in COMSOL, with emphasis on ensuring sufficient mesh resolution in key areas of the yoke to accurately capture magnetic field gradient variations. This is to avoid misjudgments regarding magnetic circuit flux leakage or local saturation due to a coarse mesh.

**Figure 9:**
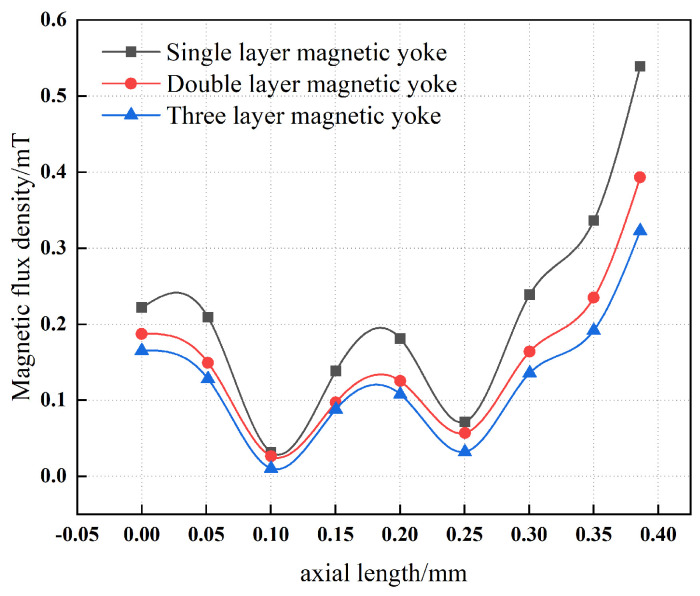
Magnetic flux density of single-layer and multi-layer magnetic yokes.

Figure 9 presents a comparison of the magnetic flux density measured at a distance of 10 mm from the surface of single-layer and multi-layer magnetic yokes. The results indicate that, with an increasing number of yoke layers, the magnetic flux density at the yoke surface decreases monotonically. This behavior demonstrates that the addition of multiple layers to the magnetic yoke effectively reduces the surface magnetic flux density and thereby enhances the overall magnetic shielding effectiveness.

#### 4.1.2. Magnetic Permeability of Single-Layer and Multi-Layer Magnetic Yokes

**Figure 10:**
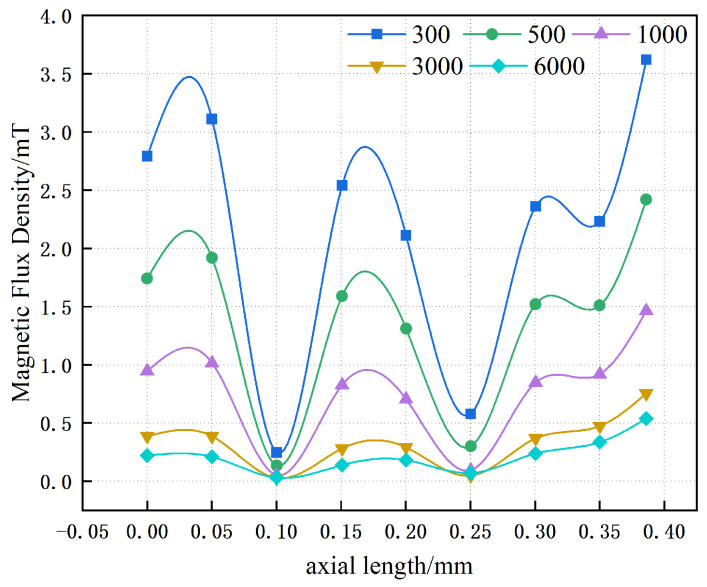
Magnetic flux density measured at a distance of 10 mm above the top surface of the single-layer magnetic yoke as a function of variations in magnetic permeability.

Figure 10 illustrates the influence of the relative magnetic permeability of a single-layer magnetic yoke on the magnitude of magnetic leakage. At low values of magnetic permeability, the magnetic flux density within the single-layer yoke is relatively high. As the permeability increases, the magnetic flux density decreases substantially, which corresponds to a pronounced reduction in magnetic leakage. Furthermore, the results indicate that when the relative magnetic permeability of the yoke exceeds approximately 3000, the magnetic flux density tends to reach a quasi-saturated regime, with the maximum magnetic flux density at a distance of 10 mm stabilizing at about 0.5 mT.

**Figure 11:**
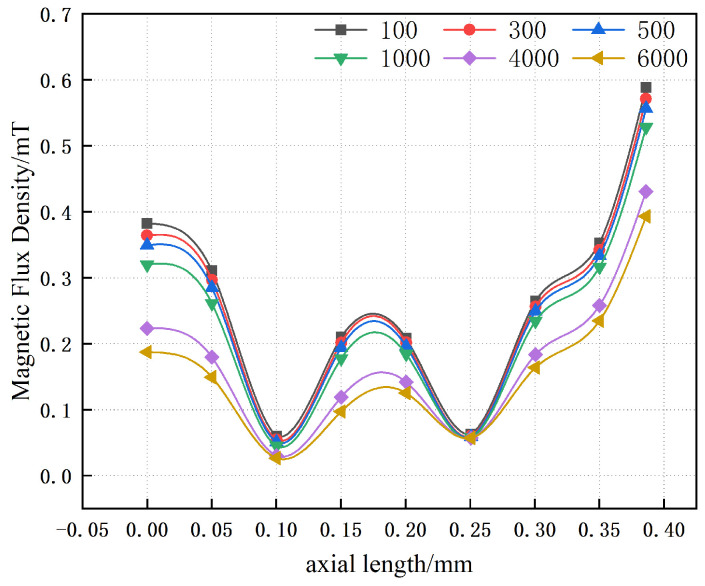
Magnetic flux density distribution on the upper surface as a function of variations in the magnetic permeability of the inner yoke.

Figure 11 illustrates the effect of varying the relative magnetic permeability of the inner yoke on the magnetic flux density, while the relative magnetic permeability of the outer magnetic yoke is held constant. It is observed that, even at comparatively low values of magnetic permeability, the maximum magnetic flux density in the multi-layer yoke remains approximately 0.55 mT. In this low-permeability regime, variations in the magnetic permeability have a negligible influence on magnetic flux leakage. Conversely, at higher values of magnetic permeability, the influence of leakage becomes increasingly significant as the permeability rises. Under these conditions, the maximum magnetic flux density at a distance of 10 mm tends to stabilize at approximately 0.4 mT.

The preceding analysis indicates that, in the regime of relatively low magnetic permeability, the magnetic flux leakage suppression capability of multi-layer yokes is markedly superior to that of single-layer yokes. In contrast, in the regime of higher magnetic permeability—for example, when the single-layer yoke has a relative permeability of 6000 and the inner and outer layers of the multi-layer configuration also each possess a relative permeability of 6000—the magnetic flux leakage suppression effect of the multi-layer yoke is improved by approximately 20% compared with the single-layer yoke.

#### 4.1.3. Influence of the Inner and Outer Yoke Layer Thicknesses on the Magnetic Core Performance

**Figure 12:**
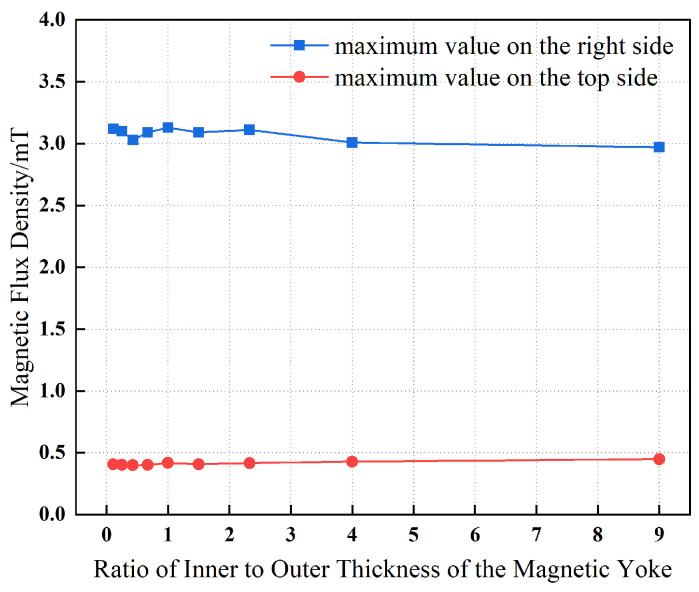
Thickness determination of inner and outer laminations of the magnetic yoke and magnetic flux density distribution on the external surface.

Figure 12 illustrates the influence of variations in the thickness of the inner layer of the magnetic yoke on the maximum magnetic flux density measured at a distance of 10 mm from the right and top surfaces, while maintaining a constant total thickness of 10 mm for the combined inner and outer yoke layers. It can be observed that, as the inner layer thickness increases from 3 mm to 7 mm, the magnetic flux density undergoes only minor variations. For the magnetic flux density near the top surface, a fluctuating behavior is observed as the inner yoke layer thickens. This behavior indicates that the magnetic shielding effectiveness of the inner yoke layer at the top surface improves with increasing thickness; however, this enhancement is non-linear and appears to be affected by the magnetic properties of the material and/or the geometric configuration of the yoke. At the right surface of the yoke, the magnetic flux density is relatively low when the inner layer thickness is small (3 mm), increases when the thickness reaches an intermediate value (5 mm), and then decreases again as the thickness is further increased. From an engineering application standpoint, the variation in the maximum magnetic flux density within the yoke induced by changes in yoke layer thickness is negligible, indicating that the relative thickness ratio of the layers is of limited significance.

### 4.2. Thickness of the Magnetic Yoke

**Figure 13:**
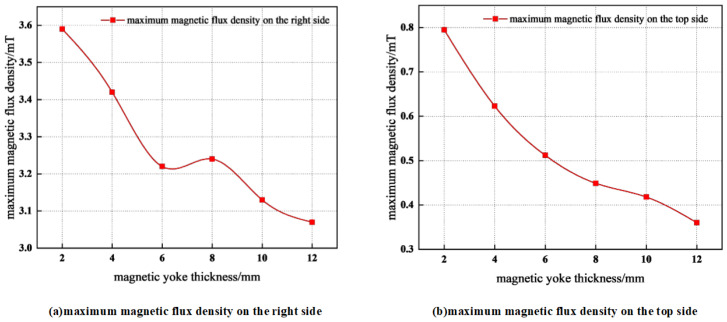
Yoke thickness and maximum magnetic flux density.

In Figure 13, it can be observed that an increase in the thickness of the magnetic yoke leads to a gradual decrease in its maximum magnetic flux density. This behavior indicates that enhancing the yoke thickness improves the magnetic circuit closure, thereby reducing magnetic flux leakage.

**Figure 14:**
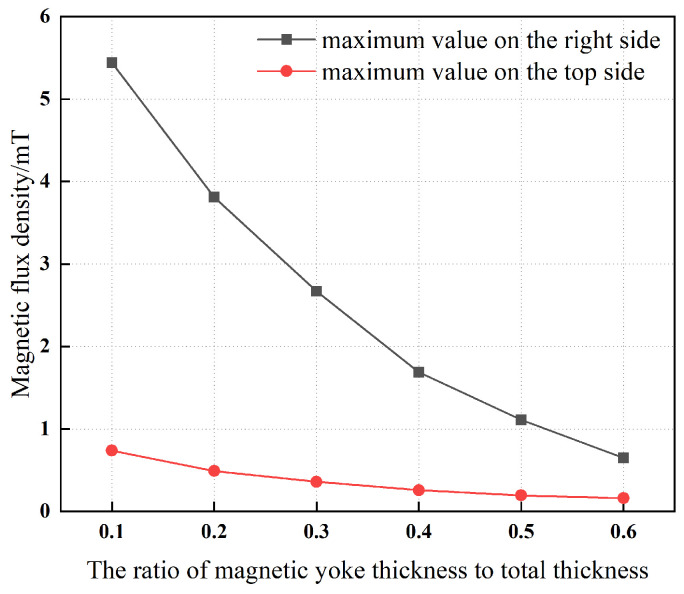
Thickness ratio and maximum magnetic flux density. The combined thickness of the magnetic yoke and the magnetic ring is maintained constant at 40 mm.

In Figure 14, with the total combined thickness of the magnetic yoke and magnetic ring constrained to 40 mm, a clear inverse relationship is observed between the proportion of thickness allocated to the magnetic yoke and the maximum magnetic flux density. This relationship indicates that increasing the thickness of the magnetic yoke mitigates magnetic leakage, thereby confirming that an appropriately increased yoke thickness can effectively improve the system’s resistance to magnetic leakage.

## 5. The Impact of Magnetic Ring Parameters on Magnetic Leakage

To establish a rigorous theoretical foundation for the final design of the permanent magnet bearing, it is necessary to analyze the influence of the magnetic ring parameters on the bearing’s performance. In this context, several critical parameters that govern the operational behavior and efficiency of the bearing are systematically investigated.

### 5.1. Number of Magnetic Rings

**Figure 15:**
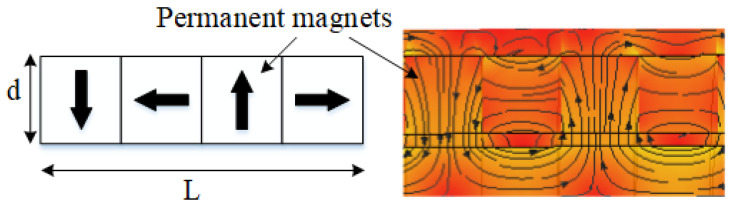
Magnetization method and magnetic flux density cloud diagram. The arrow represents the direction from the north pole to the south pole.

The number of magnetic rings in the permanent magnet array plays a critical role in determining the spatial frequency of the magnetization directions within the array. In our design, a Halbach configuration is adopted, in which each unit cell consists of four pairs of semi-circular arc-shaped magnetic rings. The magnetization scheme and the corresponding magnetic flux density distribution (cloud plot) are presented in Figure 15.

**Figure 16:**
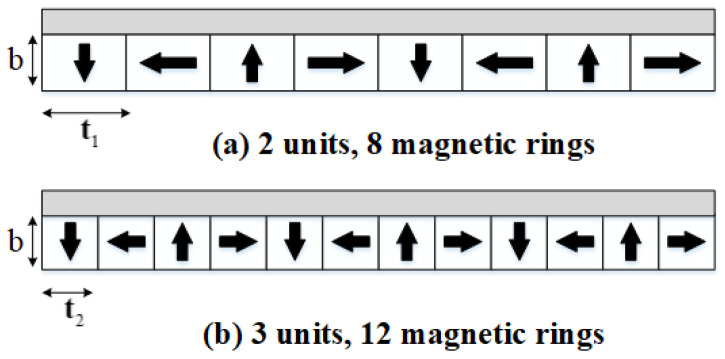
The number of units and the quantity of magnetic rings. The arrow represents the direction from the north pole to the south pole.

While the radial thickness of the magnetic rings is kept constant, increasing the number of units modifies only the axial width of these rings. Figure 16 illustrates the 2-unit and 3-unit magnetic ring configurations, in which the radial thickness *d* of the magnetic rings is maintained, whereas the axial width of an individual magnetic ring varies. In the 2-unit configuration, the thickness of a single magnetic iron element is denoted by $t_{1}$, and in the 3-unit configuration, it is denoted by $t_{2}$.

The specific relationship between the number of units and the axial width of the magnetic rings is summarized in Table 5.

**Figure 17:**
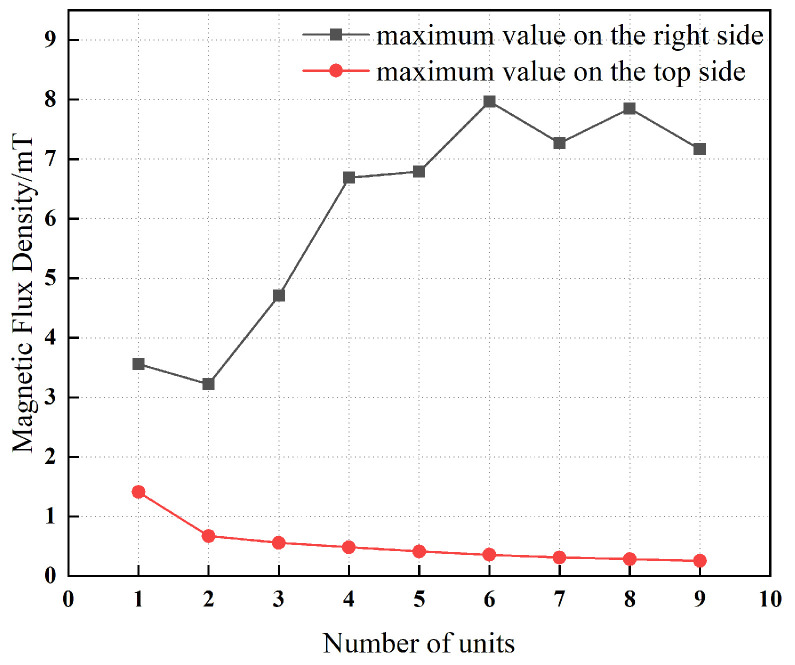
Number of units and maximum magnetic flux density.

Figure 17 demonstrates that, with an increasing number of magnetic rings, the magnetic flux density on the upper surface of the magnetic yoke decreases monotonically. At small ring counts, the unilateral magnetic field concentration effect characteristic of the Halbach array is not yet dominant; consequently, the magnetic flux density in this region is governed primarily by the contribution of the radially magnetized rings. As the number of rings increases, the Halbach configuration becomes progressively more effective, strengthening the magnetic field preferentially on one side of the array while concomitantly attenuating it on the opposite side. This asymmetric field enhancement leads to a reduction in the magnetic flux density on the top surface of the yoke. In contrast, as the number of magnetic rings increases, the magnetic flux density on the right-hand surface of the yoke exhibits an overall increasing trend. This behavior is attributed to the cumulative interaction among the magnetic rings within the Halbach configuration, which collectively reinforces the magnetic field in this region and yields a net increase in magnetic flux density. Examination of the curves in Figure 17 indicates that, when the number of units is 2 or 3 (corresponding to 8 or 12 magnetic rings), the peak magnetic flux density on both the right and upper surfaces is comparatively low. This result implies that these configurations realize a more favorable compromise between the spatial distribution of the magnetic field and the magnitude of the magnetic flux density.

### 5.2. Arc Radius of the Magnetic Ring

**Figure 18:**
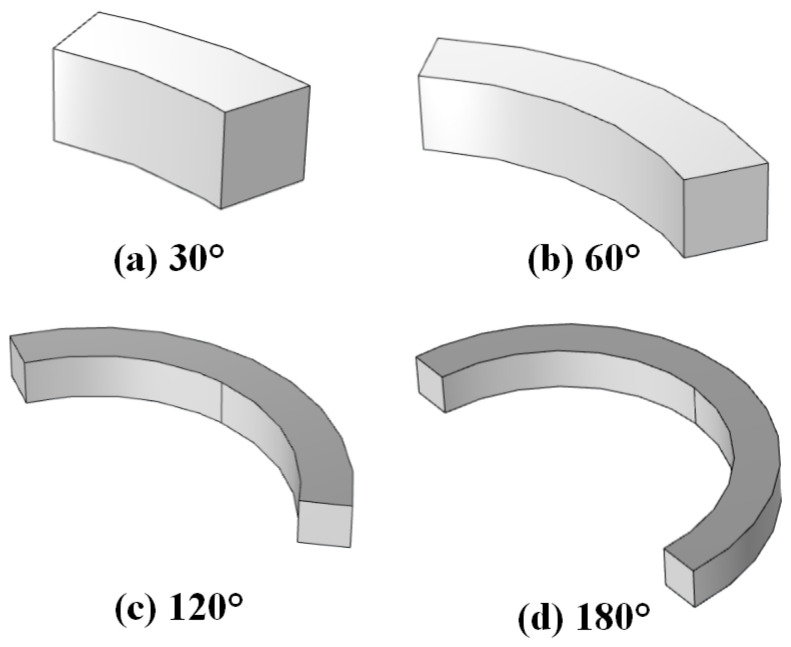
Magnetic rings with different arc angles.

The arc radius of the magnetic ring is a key parameter in determining the volume of the permanent magnet and, consequently, exerts a direct influence on the resulting magnetic flux density. Figure 18 depicts magnetic rings with different central angles, in which the inner and outer diameters are held constant while only the magnitude of the subtended angle (radian measure) is varied.

**Figure 19:**
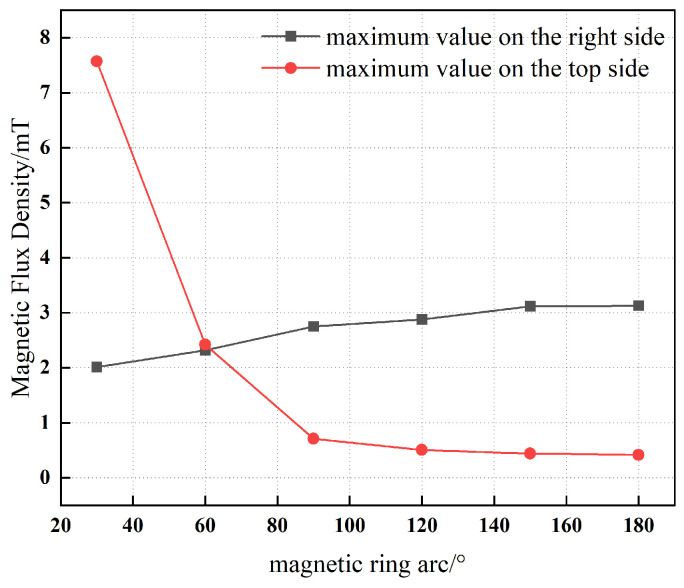
Arc of magnetic rings and maximum magnetic flux density.

Figure 19 presents the variation in the maximum magnetic flux density on the right and upper surfaces of the magnetic yoke as the arc radius of the magnetic ring increases from 30° to 180°. As the arc radius increases, the maximum magnetic flux density on the upper surface of the yoke decreases markedly, whereas the corresponding value on the right surface exhibits a monotonic increase. This behavior is attributed to the redistribution of magnetic flux induced by geometric modifications of the magnetic ring.

**Figure 20:**
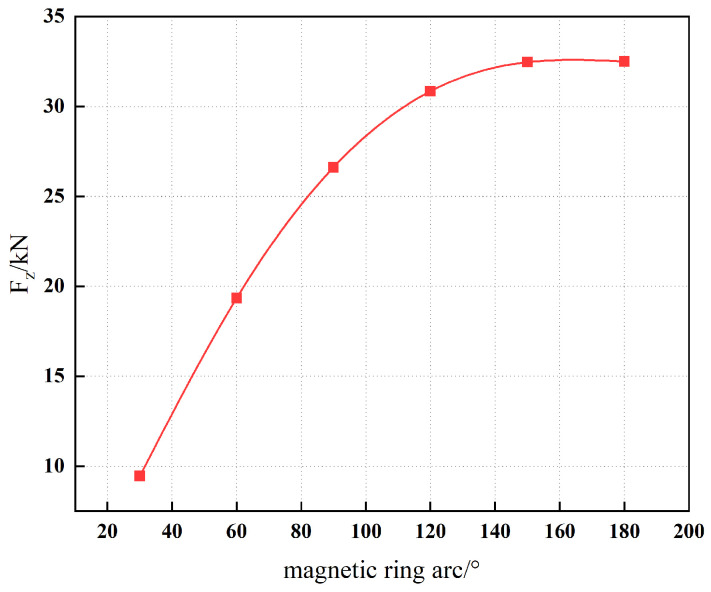
Magnetic ring arc and magnetic force.

In addition, Figure 20 illustrates the dependence of the magnetic force on the arc radius. The results indicate that an arc angle in the range of 150° to 180° is preferable for the magnetic ring design. Within this interval, the magnetic field distribution along the yoke is more uniform, thereby enhancing the operational performance and stability of the permanent magnet sliding bearing.

### 5.3. Structural Comparison

**Figure 21:**
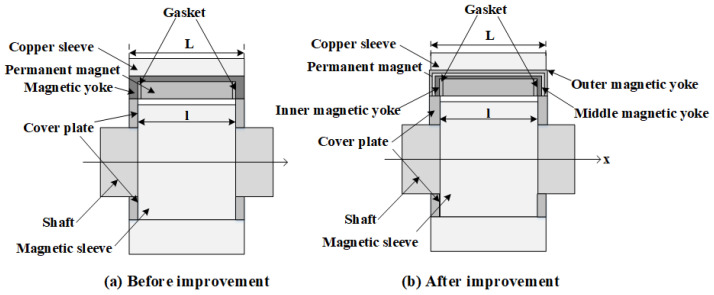
Improved permanent magnet bearing structure.

Building upon the preceding analysis, the influence of yoke thickness, the implementation of multi-layer yokes, and the optimization of magnetic ring parameters on magnetic leakage performance has been systematically investigated. Consequently, substantial improvements have been introduced to the original structural configuration, as illustrated in Figure 21. In the optimized design, the total yoke thickness is fixed at 12 mm, with the inner yoke layer having a thickness of 6 mm, while the middle and outer yoke layers are each configured with a thickness of 3 mm. In addition, a set of 12 magnetic rings, each with a curvature of 180°, has been employed.

**Figure 22:**
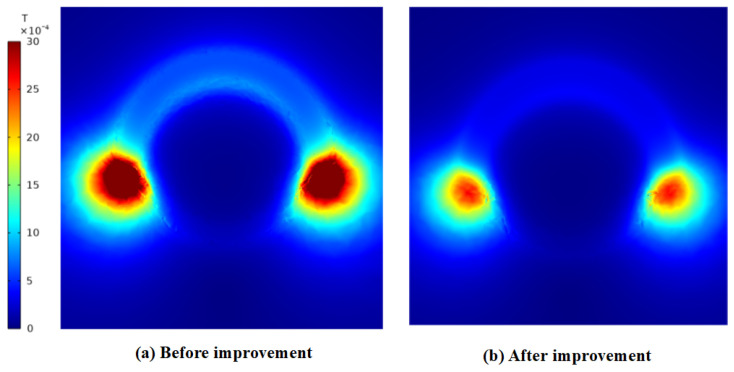
The maximum magnetic flux density on the right side before and after the improvement. (**a**) Initial design, 4.71 mT (**b**); after refinement, 2.71 mT.

**Figure 23:**
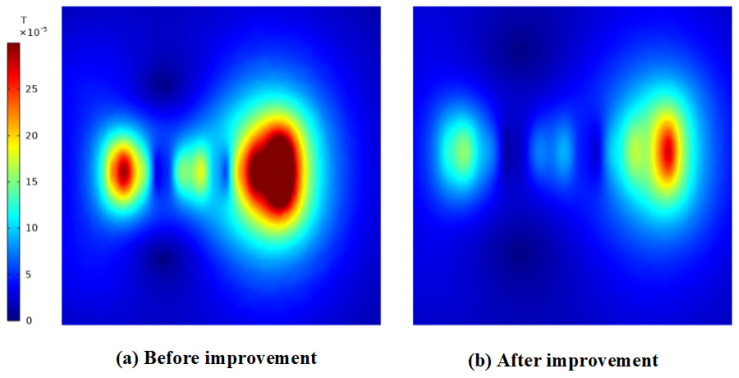
The maximum magnetic flux density on the top side before and after refinement. (**a**) Initial design, 0.56 mT (**b**); after refinement, 0.27 mT.

The magnetic flux density distribution of the original configuration is shown in Figure 22a and Figure 23a, exhibiting maximum magnetic flux density values of 4.71 mT on the right side and 0.56 mT on the top side, respectively. In contrast, the corresponding distribution for the optimized structure is presented in Figure 22b and Figure 23b, where the maximum magnetic flux density values are reduced to 2.71 mT on the right side and 0.27 mT on the top side. This comparison demonstrates a substantial enhancement in the mitigation of magnetic flux leakage in the optimized structure relative to the original design.

A comprehensive simulation analysis is conducted focusing on the radial magnetic force, which is the key indicator of load-bearing capacity. The arc angle of the magnetic ring was identified as a critical parameter, as it determines the volume of the permanent magnet and the effective area of the air gap between the Halbach array and the magnetic sleeve.

**Figure 24:**
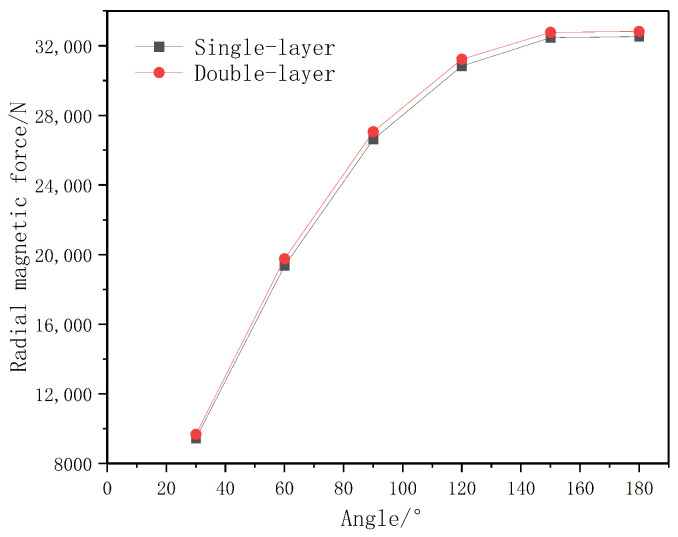
Comparison of radial magnetic forces generated by single-layer and double-layer yoke structures across varying magnetic ring arc angles.

We have performed a comparative analysis of the radial magnetic force for both single-layer and double-layer yoke configurations across different arc angles while keeping the inner and outer diameters constant, as shown in Figure 24. The results reveal that, as expected, the radial magnetic force increases monotonically as the arc angle of the magnetic ring increases. Contrary to the concern that the multi-layer structure might reduce performance, the simulations indicate that the double-layer yoke actually generates a slightly higher radial force compared to the single-layer yoke under identical conditions.

## 6. Conclusions

This study has verified the effectiveness of a multi-layer yoke structure in suppressing magnetic leakage in permanent magnet sliding bearings. Based on comprehensive finite element simulations, it has been demonstrated that design measures such as optimizing the number and angular span of magnetic rings, as well as adjusting the yoke thickness, can significantly enhance the leakage-resistant characteristics of the system. The substitution of a conventional single-layer yoke with an optimized multi-layer configuration leads to a pronounced reduction in magnetic leakage without degrading the bearing’s load-carrying capacity.

The analysis of the magnetic flux density distribution further confirms that increasing the number of yoke layers effectively reduces the surface magnetic flux density and improves the magnetic shielding performance. In particular, under conditions of higher magnetic permeability, the multi-layer yoke configuration exhibits superior leakage suppression relative to the single-layer design, achieving an improvement in leakage prevention exceeding 20%. Engineering application analyses indicate that, although the thickness ratio among individual yoke layers exerts only a minor influence on the maximum magnetic flux density, the overall performance of the leakage-proof design is substantially enhanced through the implementation of multi-layer yoke structures.

## Figures and Tables

**Table 1:** Structural parameters of the conductive rotor.

Structure Name	Parameter	Symbol	Numerical Value
Magnetic Sleeve	Inner Diameter/mm	$R_{1}$	95
	Outer Diameter/mm	$R_{2}$	115
	Axial Width/mm	l	366
Driveshaft	Radius/mm	$R_{1}$	95

**Table 2:** Structural parameters of stator.

Structure Name	Parameter	Symbol	Numerical Value
Magnetic Ring	Inner Diameter/mm	$R_{3}$	120
	Outer Diameter/mm	$R_{4}$	150
Magnetic Yoke Iron	Outer Diameter/mm	$R_{5}$	160
	Radial Thickness/mm	–	10
	Axial Width/mm	L	386
Gasket	Inner Diameter/mm	$R_{3}$	120
	Outer Diameter/mm	$R_{4}$	150
	Axial Width/mm	–	3

**Table 3:** Structural dimensions of the calculated magnetic bearing.

Structure	Parameter	Symbol	Value (mm)
Magnetic Sleeve	Inner Diameter	$R_{1}$	95
Magnetic Sleeve	Outer Diameter	$R_{2}$	115
Magnetic Sleeve	Axial Width	*l*	366
Rotating Shaft	Radius	$R_{1}$	95
Magnet Ring	Inner Diameter	$R_{3}$	120
Magnet Ring	Outer Diameter	$R_{4}$	150
Yoke	Outer Diameter	$R_{5}$	160
Yoke	Radial Thickness	–	10
Yoke	Axial Width	*L*	386
Spacer	Inner Diameter	$R_{3}$	120
Spacer	Outer Diameter	$R_{4}$	150
Spacer	Axial Width	–	3

**Table 4:** Material properties of the calculated magnetic bearing.

Structure	Material	Relative Permeability ($\mu_{r}$)
Yoke	Soft Magnetic Material—Soft Iron	6000
Spacer	Aluminum	1
Magnetic Sleeve	Iron	4000
Copper Shell	Copper	1

**Table 5:** Number of units and axial width.

Number of Units	Number of Magnetic Rings	Width of Unit (mm)
1	4	90
2	8	45
3	12	30
4	16	22.5
5	20	18
6	24	15
7	28	12.85
8	32	11.25
9	36	10

## Data Availability

The original contributions presented in this study are included in the article. Further inquiries can be directed to the corresponding author.

## References

[1] Kasarda M. (2000). An Overview of Active Magnetic Bearing Technology and Applications. Shock Vib. Dig..

[2] Samanta P., Hirani H. (2022). On the Evolution of Passive Magnetic Bearings. J. Tribol..

[3] Belguerras L., Mezani S., Lubin T. (2021). Analytical Modeling of an Axial Field Magnetic Coupler with Cylindrical Magnets. IEEE Trans. Magn..

[4] Fang J., Le Y., Sun J., Wang K. (2012). Analysis and Design of Passive Magnetic Bearing and Damping System for High-Speed Compressor. IEEE Trans. Magn..

[5] Wang N., Hu Y., Wu H., Zhang J., Song C. (2013). Research on Forces and Dynamics of Maglev Wind Turbine Generator. J. Magn..

[6] Lijesh K.P., Hirani H. (2015). Modeling and Development of RMD Configuration Magnetic Bearing. Tribol. Ind..

[7] Okamoto E., Ishida Y., Yano T., Mitamura Y. (2014). Passive magnetic bearing in the 3rd generation miniature axial flow pump-the valvo pump 2. J. Artif. Organs Off. J. Jpn. Soc. Artif. Organs.

[8] Jo J.H., Ryu Y.G., Choe Y. (2023). Simulation on modified multi-surface levitation structure of superconducting magnetic bearing for flywheel energy storage system by H-formulation and Taguchi method. Phys. C Supercond. Its Appl..

[9] Tanya K.K., Anirudh E., Dipak K.G. (2025). Attitude control of Earth-pointing satellites employing novel hybrid actuator configurations. Aerosp. Sci. Technol..

[10] Paul G., Rezaienia M., Rahideh A., Munjiza A., Korakianitis T. (2016). The Effects of Ambulatory Accelerations on the Stability of a Magnetically Suspended Impeller for an Implantable Blood Pump: Ambulatory Stability of Magnetic Suspension. Artif. Organs.

[11] Wang Z., Hu K., Guo Y., Wang S. (2018). Optimization on detent force characteristics of the permanent magnet suspension belt conveyor. Adv. Mech. Eng..

[12] Yonnet J.P. (1978). Passive magnetic bearings with permanent magnets. IEEE Trans. Magn..

[13] Yonnet J.P., Lemarquand G., Hemmerlin S., Olivier R.E. (1991). Stacked structures of passive magnetic bearings. J. Appl. Phys..

[14] Samanta P., Hirani H. (2008). Magnetic Bearing Configurations: Theoretical and Experimental Studies. IEEE Trans. Magn..

[15] Bekinal S.I., Doddamani M. (2020). Friction-Free Permanent Magnet Bearings for Rotating Shafts: A Comprehensive Review. Prog. Electromagn. Res. C.

[16] Haidl P., Buchroithner A. (2021). Design of a Low-Loss, Low-Cost Rolling Element Bearing System for a 5 kWh/100 kW Flywheel Energy Storage System. Energies.

[17] Dagnaes-Hansen N.A., Santos I.F. (2019). Permanent magnet thrust bearings for flywheel energy storage systems: Analytical, numerical, and experimental comparisons. Proc. Inst. Mech. Engineers. Part C J. Mech. Eng. Sci..

[18] Li S., Yang X. (2022). Research on Static Performance of Ship Magnetic Composite Water-lubricated Rubber Stern Bearing. Ship Sci. Technol..

[19] He T., Wang J., Yao S., Shi J., Zhang Q., Xu H., Zhao B. (2022). Tribological and Dynamic Performances of Composite Water Lubricated Bearing with Magnetic Support. J. Propuls. Technol..

[20] Li Z., Ouyang W., Wang S., He T., Wang B. (2022). Simulation of the carrying capacity of the new type water-lubricated magnetic-hydraulic double suspension stern bearing. J. Ship Mech..

[21] Wang S., Ouyang W., Li Z., Wang B. (2021). Load carrying capacity of a novel magnetic-liquid double suspension fixed pad thrust bearing. Ind. Lubr. Tribol..

[22] Wang B., Ouyang W., Wang S., Sheng C., He T., Yan Z. (2023). Load Carrying Capacity Enhancing Design and Lubrication Investigation of the Magnetic-Water Double Suspension Elastic Support Thrust Bearing. Lubricants.

[23] Zhou J., Fang Z., He S., Zhang Q. (2024). Modelling and stability analysis of the permanent magnetic bearing-rotor system under base excitation. Arch. Appl. Mech..

[24] Chen Z., Wang J., Li R., Liu Y. (2024). Study on the transient performance of textured water-lubricated bearings considering different acceleration conditions. Phys. Fluids.

[25] Cheng W., Xue S., Xiao L., Feng S., Xu G. (2025). Load-Bearing Characteristics of Permanent Magnetic-Foil Hybrid Bearings. Bearing.

[26] Ge Z., Mei L. (2024). Novel Modular Permanent Magnet-Biased Magnetic Bearing: Structural Design and Magnetic Field Analysis. Bearing.

[27] Cheng W., Wang Y., Bu C., Xiao L., Feng S., Xu G. (2025). Research on Load-Bearing Characteristics and Experiments of Radial Passive Permanent Magnetic Bearings. J. Mech. Eng..

[28] Supreeth D.K., Siddappa I.B., Shivamurthy R.C. (2025). Double flux radial electrodynamic bearing with the radial air gap between permanent magnet rings and its optimization using artificial neural network for bearing characteristics. Eng. Res. Express.

[29] Margarit G., Charalampos S., Stefanos G. (2024). Towards Production of Cost-Effective Modification of SmCo 5 -Type Alloys Suitable for Permanent Magnets. Materials.

[30] Amit M., Sina K., Tomaž T., Benjamin P., Sašo Š., Carlo B., Kristina Ž. (2023). Short-Loop Recycling of Nd-Fe-B Permanent Magnets: A Sustainable Solution for the RE 2 Fe 14 B Matrix Phase Recovery. Materials.

[31] Tomaž T., Pierre K., Rosario M.L., Peter F., Laura G., Matej Z., Carlo B. (2024). Magnetic Performance and Anticorrosion Coating Stability of Thermally Demagnetized Nd-Fe-B Permanent Magnets for Reuse Applications. Materials.

[32] International Commission on Non-Ionizing Radiation Protection (1998). Guidelines for limiting exposure to time-varying electric, magnetic, and electromagnetic fields (up to 300 GHz). Health Phys..

[33] International Commission on Non-Ionizing Radiation Protection (2009). Guidelines on limits of exposure to static magnetic fields. Health Phys..

[34] (2002). IEEE Standard for Safety Levels with Respect to Human Exposure to Electromagnetic Fields, 0–3 kHz.

[35] Contessa G.M., D’Agostino S., Falsaperla R., Grandi C., Polichetti A. (2021). Issues in the Implementation of Directive 2013/35/EU Regarding the Protection of Workers against Electromagnetic Fields. Int. J. Environ. Res. Public Health.

[36] Cheng X., Liu W., Sun M., Luo W. (2021). Research on Transmitted Torque Calculation Method of Permanent Magnet Eddy-Current Couplers. J. Northeast. Univ. (Nat. Sci.).

[37] Yuan K.P., Zhang G., Xie C.Q., Song X. (2019). Integral definition method to solve magnetic force of axial permanent magnetic bearing. IOP Conf. Ser. Mater. Sci. Eng..

[38] Tian L.L., Ai X.P., Tian Y.Q. (2012). Analytical Model of Magnetic Force for Axial Stack Permanent-Magnet Bearings. IEEE Trans. Magn..

[39] Kim H.E., Kim K., Ma T.Y., Kang T.G. (2017). Numerical investigation of the dynamics of Janus magnetic particles in a rotating magnetic field. Korea-Aust. Rheol. J..

[40] Pile R., Le M.Y., Le B.J., Parent G. (2020). Study of the Combined Effects of the Air-Gap Transfer for Maxwell Tensor and the Tooth Mechanical Modulation in Electrical Machines. IEEE Trans. Magn..

